# Severe Acute Kidney Injury Associated with *Giardia lamblia* Infection

**DOI:** 10.4269/ajtmh.23-0075

**Published:** 2023-05-01

**Authors:** Kazuhiro Ishikawa

**Affiliations:** Department of Infectious Diseases, Tokyo Medical University Ibaraki Medical Center, Ibaraki, Japan

A 52-year-old man developed watery diarrhea occurring roughly 10 times daily 2 weeks prior to admission. Because his symptoms did not resolve, he visited a local clinic 8 days prior to admission. His serum creatinine and blood urea nitrogen were found to be 5.1 and 78.9 mg/dL at the clinic, respectively. He received normal saline intravenously daily; however, his renal function did not improve. The patient was subsequently admitted to our hospital. He had no prior infections, no travel history, no contact with sick individuals, no history of consuming raw food, no exposure to feces, no neonatal contact, and no same-sex sexual partners. He did, however, have a history of oral–anal intercourse with a Japanese female sex worker 1 month prior to admission.

**Figure 1.**
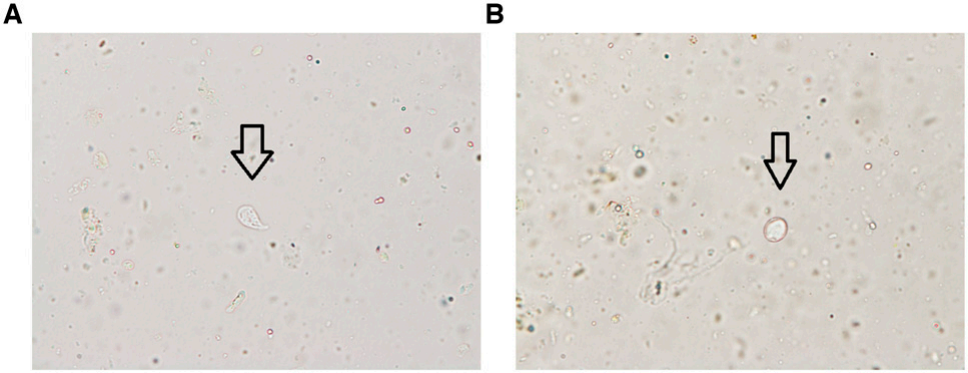
The smear of a lavage of a colon fiber revealed a trophozoite (left) and cyst (right).

On admission, his vital signs were as follows: Glasgow Coma Scale, E4V5M6; temperature, 36.5°C; blood pressure, 111/82 mm of Hg; and pulse rate, 123 beats per minute. Laboratory test results revealed the following: white blood cell count, 19,900/μL (neutrophils, 81.0%); hemoglobin, 18.3 mg/dL; platelet count, 455 × 10^3^/μL; blood urea nitrogen, 38.6 mg/dL; creatine, 4.0 mg/dL; and C-reactive protein, 0.5 mg/dL. His HIV test was negative. Urinalysis results were as follows: sodium, 32 mEq/L; potassium, 27 mEq/L; and chloride, 59 mEq/L. An abdominal computed tomography (CT) scan and colonoscopy confirmed atypical findings. A colon biopsy revealed mild inflammation. The bowel lavage smear was culture negative. Microscopic examination observed trophozoites and *Giardia lamblia* cysts in the stool (Figure 1). Stool organism cultures (including for *Shigella*) were negative. We suspected that *G. lamblia* from the female anus was transmitted to the patient via the fecal–oral route. The patient was treated accordingly with oral 500 mg metronidazole (three times per day for 8 days) until his next outpatient clinic visit (generally 5–7 days afterward for *G. lamblia*). His diarrhea ameliorated quickly, and his creatinine level improved to 0.76 mg/dL. A direct stool smear on day 46 was negative for *G. lamblia*.

A trophozoite is a parasite on the mucosa of the duodenum, the upper jejunum, and sometimes the gallbladder and bile ducts. *Giardia* causes disease without penetrating the epithelium, invading the surrounding tissues, or entering the bloodstream.^1^ It cannot be diagnosed using a CT scan or colonoscopy. A stool smear needs to be evaluated if trophozoites are suspected.

*Giardia* spreads easily between people, and a small amount of *Giardia* can cause illness. Because *Giardia* are found in feces, anything contaminated by stool can spread the organism.

*Giardia* can survive in stool for several weeks, so patients should refrain from sex (vaginal, anal, and oral) for several weeks after resolution of the diarrhea or after treatment. Furthermore, frequent handwashing can prevent spread and autoinfection, especially during periods of high contagiousness. Antigen detection assays, nucleic acid detection assays, and stool microscopy are all diagnostic tools for giardiasis.^2^ Stool microscopy is less sensitive than antigen and nucleic acid detection testing.

From 2012 to 2017 in the United States, public health officials (from 26 states) reported 111 giardiasis outbreaks including 760 primary cases, 28 hospitalizations, 48 emergency department visits, and no deaths.^3^

Because the renal injury in our case showed rapid alleviation with only hydration, prerenal renal failure due to dehydration is considered. Acute kidney injury due to severe diarrhea caused by *Giardia* is very rare; we were able to find only one case of coinfection with *Salmonella paratyphi* A.^4^ However, there have been reports of infection-related interstitial nephritis.^5^ If the patient does not respond to hydration therapy, a renal biopsy should be considered.^2^
